# Barriers and facilitators for transitioning of young people from adolescent clinics to adult ART clinics in Uganda: unintended consequences of successful adolescent ART clinics

**DOI:** 10.1186/s12913-020-05701-9

**Published:** 2020-09-05

**Authors:** Scovia Nalugo Mbalinda, Sabrina Bakeera-Kitaka, Derrick Amooti Lusota, Eleanor Namusoke Magongo, Philippa Musoke, Dan Kabonge Kaye

**Affiliations:** 1 Department of Nursing, College of Health Sciences, Makerere University, Kampala, Uganda; 2 Department of Pediatrics, College of Health Science, Makerere University, Kampala, Uganda; 3 Pediatric HIV Care & Treatment at AIDS Control Program, Ministry of Health, Kampala, Uganda; 4 Department of Obstetrics and Gynaecology, College of Health Science, Makerere University, Kampala, Uganda

**Keywords:** HIV, Adolescents, Transitioning, Facilitators, Barriers, ART clinics

## Abstract

**Background:**

There is a growing number of adolescents and young adults living with HIV (YPLHIV) who require the transfer of care from pediatric/ adolescent clinics to adult Antiretroviral therapy (ART) clinics. A successful transition is critical for optimum health outcomes, yet facilities may lack infrastructure, human resources (with appropriate knowledge and skills), and a supportive environment, as only 3% of clinics in Uganda caring for YPLHIV have a process for supporting this critical transition from pediatric to adult care, and, facilitators and barriers of a successful transition are not well documented. The purpose of this study was to explore the facilitators and barriers of transitioning among adolescents from adolescent clinics to adult ART clinics.

**Method:**

Eighteen focus group discussions were held in nine health facilities with 174 adolescents and YPLHIV to assess barriers and facilitators regarding transitioning to adult clinics. The focus group discussions were audio-recorded and transcribed. The Silences Framework using a thematic approach guided the analysis.

**Results:**

The key emerging issues were: Unfriendly adults in adult clinics, Care provided in the adolescent clinics, fear of stigma from health care providers, Congestion and long waiting time, fear to lose friends were barriers to transitioning. Transitioning preparation is key to a successful transition**,** moving as a cohort facilitates transition, and care in adult clinics offers new opportunities, could facilitate readiness and transition.

**Conclusion:**

YPLHIV expressed fear to transition to adult clinics mainly because of the perceived better care provided in the adolescent clinic, thus constituting a barrier to smooth transition A range of individual, social and health system and services-related factors hindered transitioning. The expectation of transitioning as a group, assurance of similar care as in the adolescent clinic, and guarantees of confidentiality, privacy, and autonomy in decision-making for care was perceived as facilitators. Understanding barriers and facilitators can enable the Ministry of Health to improve the quality of life of YPLHIV through linkage to care, adherence, retention, and viral suppression. There is a need to better planning and preparation for clinical providers and YPLHIV with a focus on age-appropriate and individualized case management transition as well as focus on improving both clinical and psychosocial support throughout the process**.**

## Background

Despite progress in reducing perinatal HIV transmission, there is a growing number of young people with HIV/AIDS (YPLHIV) who will require the transfer of care from pediatric to adult providers [1]. According to the United Nations Population Fund (UNFPA, 2017) 1.5 million Ugandans currently live with HIV. HIV prevalence among adults 15–49 years is 8.3% with HIV prevalence of youth age 15–24 at 3.2% (female) and 1.9% (male) [2]. HIV-infected adolescents (10–19 years) and young adults (20–24 years) are an increasing proportion of the HIV-infected population in Uganda [3], with over 110,000 HIV-infected adolescents in Uganda in 2012 [4]. It is estimated that 567 young people aged 15–24 years old are infected with HIV every week (Government Citizen’s Interaction Centre [GCIC], 2017). HIV prevalence is four times higher among young women compared to young men [5] increasing the risk for perinatal transmission. There is a growing number of adolescents and young adults with HIV who require the transfer of care from pediatric to adult providers; yet, globally, many young people experience barriers (e.g., infrastructure, staff training) that complicate this process [1, 6, 7].

Transition is “a planned process by which HIV-infected adolescents and young adults, and their caregivers, are empowered with knowledge and skills to enable them to independently manage their health” [8]. The main goal of transitional care is for YPLHIV to develop confidence, autonomy, and responsibility for their HIV care by the time they are required to attend adult clinics. A good transition process builds life-skills and reduces risk-taking behaviors that can interfere with adherence to treatment and retention in care. Additionally, keeping YPLHIV on ART continuously and preventing transmission to others is critical to any transition program [1, 9].

Attention to this transition is critical to ensure continuity of complex care and can help mitigate potential adverse physical and psychological complications resulting from HIV-infection or the use of long-term therapies. Adolescent transition outcomes have been reported in high- and middle-income countries in North America, Europe, and Asia [5, 10–14]. These studies do not reflect the challenges of HIV care in sub-Saharan Africa, given that limited-resources increases the complexity of transition and its evaluation. Asire and colleagues assessed both private and public clinics in Uganda caring for YPLHIV and found that only 3% of healthcare facilities had a specific health transition clinic (HTC) to support the transition from pediatric providers to adult providers [7]. Additionally, Nyabigambo and colleagues found that HTC use is less common in those who are older (age 20–24), male, live in rural locations, acquired HIV behaviorally, are not on antiretroviral therapy (ART), and have CD4 counts > 250 [9]. Therefore, those at the highest risk for health complications and transmission of HIV to others do not have access to HTC resources to support a successful transition. Efforts have been made by the Ministry of Health to expand the 3% availability to 100% so that the Transitioning process is prioritized at a National Level. The Young Adolescent Programme Support (YAPS) Project which started in 2019 is attempting to empower adolescents and young people to contribute to the reduction of HIV related morbidity and mortality among adolescents and young people living with HIV, increasing the proportion of adolescents and young people living with HIV who know their HIV status, improve ART treatment coverage of adolescents and young people living with HIV, increase viral load suppression among adolescents and young people living with HIV, and strengthen psychosocial care and support services for AYPLHIV to cope better with their HIV status [15].

In high-income countries, the transition from pediatric to adult care services usually involves a change reflects a broader gap in bridging from pediatric- and adolescent- to adult-oriented services for of clinic and /or providers, largely because care is provided as a specialized service and transitional models of care are available [16, 17]. The lack of structured transition programs reflects a broader gap in bridging from pediatric- and adolescent- to adult-oriented services for chronic illness in general, and for HIV care specifically [1]. The model of care in sub-Saharan Africa is predominantly non-specialist, comprising integrated clinics where adults, adolescents, and children living with HIV are seen by the same staff in the same place [1]. However, adolescent-friendly services, usually in the form of a dedicated adolescent clinic day, are increasingly being implemented [1, 13]. Although specific services that address the unique needs of adolescents may be available, practices vary in many of the clinics.

Assessment of adolescent HIV care found “the need for better planning and preparation for clinical providers and adolescents to improve the transition process, with a focus on improving both clinical and psychosocial support throughout the process” [1]. However, there is no clear process of transitioning to adult care in Ugandan health clinics. The purpose of this study was to explore the facilitators and barriers of transitioning among adolescents from adolescent clinics to adult ART clinics. Ensuring effective transition from pediatric/adolescent to adult care is a national priority for optimizing the health of YPLHIV, and also critical for the prevention of HIV transmission to wider communities.

## Methods

### Setting and participants

The study was conducted from August 2019 to January 2020 in 9 facilities at various health care system levels; 3 Regional hospitals, 2 District hospital 2 Health Center IV, and 2 HC III. The sample was drawn across the health care system because all the facilities are involved in testing and treating HIV and they receive participants across all socio-economic levels. The facilities also represent all the regions of the country. Data was collected through 18 focus group discussions with young people living with HIV in nine facilities.

### Data collection and analysis

An explorative qualitative approach was utilised for this study using eighteen (18) focus group discussions. Data were collected from 9 health facilities in all the four regions of Uganda. The study participants were selected through purposeful sampling based on sex and age. Eighteen focus group discussions were held, nine for the females and nine for males with an average of 10 young people in each focus group.

These participants were identified through the peer educators who were approached at the beginning of the study. The inclusion criteria were being an adolescent or young person, being HIV positive, and attending the adolescent HIV clinic. All participants gave verbal informed consent and assent and were assured that the information given was confidential, that they were not obliged to join the study, and that their views would be anonymous. Telephone contacts of the adolescents and young people were obtained and they were informed that they would be called back again for a similar discussion.

Participants were selected purposefully by maximum variation sampling to represent a variety of age groups, education levels, and socioeconomic status. All the interviews were conducted in one of the private offices on the ward by the first Author (SNM). We explored the following issues concerning the transitioning of adolescents to adult clinics. (Responsibility of their health, Knowledge about their health, responsible behavior, the introduction of transitioning process, experiences of adolescents who had transitioned and now they were back in the adolescent clinics, facilitators and barriers in transitioning to adult clinics). The interview guide was developed using literature and the Ministry of Health (MOH) guidelines on transitioning which provided guided information and areas which are addressed in transitioning. Some of the areas explored were from the ministry of the guidelines and others were from the literature reviewed.

Interviews lasted for 60–90 min and were conducted in English and other local languages depending on the location. The proceedings were tape-recorded. At the end of each interview, the key points were summarised to the participants to verify the data. Handwritten notes were taken during interviews.

The focus group interviews were initially reviewed by the first author during data collection to assess the point of data saturation and to identify areas of further interrogation in the subsequent FGDs, as well as areas for clarification as part of the Silences Framework method of data analysis. After data collection, all the FGDs were transcribed verbatim. The transcribed text was then translated from the local language back into English. Independent researcher assistants conversant in both local languages and English cross-checked the transcripts for accuracy and language translation consistency. As the researchers aimed at exploring experiences, barriers, and facilitators to transition. The interviews were read through several times by all authors separately and the came together and discussed the different statements. The different statements were grouped, resulting in the construction of a map, following the description by Braun and Clark [18]. Different themes and sub-themes were identified and discussed and rearranged until a final pattern was distinguished. The themes that were relevant to our research questions were considered and reported.

The Silences Framework [19] guided this research. The Framework asserts that reality is not objective or fixed but rather human beings scripts the social world in communities at a particular time [19]. The Framework emphasised the ‘Screaming Silences’ in individual and group interpretations of experiences that can be qualified as ‘truth’. In this paper, silences are explored about facilitators and barriers of transitioning among the adolescents in ART clinics in 9 facilities. The issues were explored in the context of successful transition outcomes such as addressing sero-status disclosure and stigma, adherence to and continuation of lifelong ART, identification and reporting or management of drug side-effects, retention in and continuity of care, and mitigation or prevention of emotional and psychosocial challenges associated with living with a stigmatizing chronic disease.

All focus group discussions were audiotaped and transcribed verbatim and the transcribed data were subjected to the four phases of the Silences Framework shown below [19].

- **Phase 1 -** After transcription, the outputs from the focus group discussions were analysed by the researcher, and recurrent themes were identified as the preliminary findings from the study.
- **Phase 2 -** The preliminary findings from phase 1 were reviewed by some of the research participants. Reflections on the early findings from the participants were used to enhance further critique, confirming, or refuting the findings from phase 1. A discussion of the silences (Findings) was formulated. The adolescent’s contact numbers or their parent’s numbers were got from the initial meeting. They were also informed about the possibility of the need for subsequent meetings to clarify or get more detailed information about what had been discussed in the initial FGDs. The consent and assent were obtained in the initial meeting but during the subsequent meetings, they were reminded about the consent they signed initially. The participation was voluntary and most of the adolescents came back and transport refund was provided as it was stipulated in the consent form
- **Phase 3 –** A further analysis of the findings from phase 2 was undertaken in this stage by research participants (A group that mirrors the research participants but they did not take part in research). The participants in this phase were drawn from the ART clinics that had not taken part in the focus group discussion with a critical indirectly associative eye for validation of the results. The consent and assent were obtained from these adolescents.
- **Phase 4 -** Finally the researcher reflected on the findings from phase 3, revisiting, reviewing, and developing emerging themes that formed the final output of this study.

### Ethical approval

Ethical reviews and approval were obtained from the Research Ethics Committee of School of Health Sciences, College of Health Sciences at Makerere University #SHSREC REF NO: 2019–029 and Uganda National Council of Science and Technology (SS5063) Administrative clearance and permissions were also obtained from the management of each of the health facilities. Written informed consent was obtained from young people above 18 years. For adolescents below 18 years assent from the adolescents and consent from parents or guardians was obtained and assent from the adolescents. Participation was voluntary and all the interviews were conducted in private settings to ensure participant’s confidentiality.

## Results

Following transcription, coding, and analysis of the focus the following themes on barriers and facilitators to transitioning to adult ART clinics. Unfriendly adults in adult clinics, Care provided in the adolescent clinics, fear of stigma from health care providers, Congestion, and long waiting time, fear to lose friends were barriers to transitioning. Transitioning preparation is key to a successful transition**,** moving as a cohort facilitates transition, and care in adult clinics offers new opportunities. Facilitators identified were; Transitioning preparation is key to a successful transition**,** moving as a cohort facilitates transition and care in adult clinics offers new opportunities.

### Socio-demographic data

A structured interview with the young people was used to obtain data on age, gender, school status mode of HIV transmission, and living status. The majority of the respondents were aged 20–24 years, in school, female, and had got HIV through perinatal transmission. (Table 1).

**Table 1:** Socio-demographic data

Variable	No	FGD1	FGD2	FGD3	FGD4	FGD5	FGD6	FGD7	FGD8	FGD9	FGD 10	FGD 11	FGD 12	FGD 13	FGD 14	FGD 15	FGD 16	FGD 17	FGD 18
**Age**																			
15–19	49	2	3	3	2	3	2	3	3	3	3	3	4	3	2	3	2	3	2
20–24	72	4	5	5	4	4	4	4	4	5	4	4	4	4	4	3	3	4	3
Above 25	53	3	3	3	2	3	3	3	3	3	2	3	3	3	3	4	4	2	3
**Education status**																			
In school	102	5	7	6	5	5	6	5	6	6	6	6	6	5	6	6	5	5	6
Out of school	72	4	4	5	3	5	3	5	4	5	3	4	5	5	3	4	4	4	2
Sex																			
Male	84		11		8		9		10		9		11		9		9		8
Female	90	9		11		10		10		11		10		10		10		9	
**Mode of transmission**																			
Perinatal	127	8	8	9	6	8	7	7	6	8	8	8	8	8	5	7	7	6	3
Horizontal	39	1	3	2	1	2	2	3	2	3	1	2	2	1	4	2	2	2	4
Don’t know	8				1				2				1	1		1		1	1
**Living status**																			
Alone	30	1	1	2	2	2	1	2	1	4	2	2	2	1	1	2	1	2	1
Parents	52	3	3	2	4	2	3	2	3	3	2	2	5	3	3	3	3	3	3
Guardian	92	5	7	7	2	6	5	6	6	4	5	6	4	6	5	5	5	4	4
**Time spent (Min)**		**72**	**75**	**80**	**60**	**80**	**83**	**75**	**80**	**90**	**78**	**80**	**87**	**85**	**78**	**80**	**65**	**80**	**68**

### Barriers to transitioning

#### Unfriendly adults in the adult ART clinics

There was an attempt to transition the adolescents to adult clinics but most of them came back to adolescent clinics. The adolescents stated that one of the barriers for them to transition to adult clinics is the judgmental, unfriendly nature of the adults in the clinics. The adolescents found it hard to talk to adults because they are so serious and talk adult stuff, they feared to be discriminated against them

*“…. the adult people are so judgemental, you hear them saying, “how did he get the HIV? such a young child! yet sometimes, you got it from your mother, like me I got it from my mother” (*female, 15-19 years).

Another fear adolescents perceive in adult HIV clinics is intentional or unintentional disclosure of HIV status. In the paediatric clinics, providers know about the adolescents’ history and therefore do not mention or ask about how the adolescents acquired HIV. Interaction with new providers in the adult HIV clinics could unintentionally lead to disclosing HIV status, but worse, could lead to stigma and/or discrimination if the HIV status is revealed to other people outside the ART clinic. This view is exemplified by two respondents:

*….and they don’t end only here, they again take them to the community and the whole village knows and then you reach there when everyone has known”. (male, 20–24 years)*

“…. *when you go to the adult clinic, it may be so difficult to comfortably associate with the adults. So, it may not be easy for us. They have parental thoughts, yet for me, I have adolescent thoughts. I don’t know if there are adults, that I will be able to converse with like it is here. So, I think it may be so hard for me to comfortably converse with them or fit in them. But maybe if I get a child, I will be able to fit in them knowing am a fellow parent”. (Female,20–24 years)*

### Care provided in adolescent clinics

Adolescents have been in adolescent clinics since they were 10 years. They have developed a routine, found friends and, care in the adolescent clinic is different and it favors them and they like it. A typical adolescents clinic starts with a reminder from the peer a day before the clinic. Those who confirm will be expected to attend the clinic and those who are not able reasons are given and if it is within the reach of the facility they are facilitated like transport.

*“….at a certain point, it comes back to the health workers. Health workers tend to treat adolescents and young people in a different way while in the adolescent clinic and therefore the adolescents don’t wish at any one point to leave their clinic to go to the adult clinic where they will not be treated the same way”. (female, above 24 years)*

The explanation for this perception was obtained from further interaction with the adolescents (Phase 2 of the Silences framework), where adolescents described the activities they go through on a typical clinic day in the adolescent HIV clinic*.* On ta real day, they start with education sessions either from the peer, health providers, or counselors depending on the schedule and experience. After the session, if they are exhausted, adolescents are fast-tracked to the pharmacy and send a maximum of 30 min. If they are not exhausted, the adolescents are taken to the counselors and then to the clinician and finally to the Pharmacy for a refill. Healthcare providers avail porridge and a bite every time they come to the clinic, they do hold psychosocial events quarterly for all the adolescents mainly to share experience, have talks, dance, eat, and play. They also have health education sessions with peers. The adolescents felt that they been favored in this adolescent clinic which they know won’t happen in the adult ART clinics, especially since some had prior experiences of the adult HIV clinic. Another reason for the preference of the care environment of the adolescent clinic was perceived confidentiality from the healthcare providers.

*“…like another reason why we might be scared to leave this adolescent clinic, we think that our clinic is more confidential and secure than the adult clinic because we feel like our secrets are safe in the adolescent clinic than in the adult clinic.” (Female,20–24 years)*

Besides, the healthcare providers tended to treat the adolescents uniformly, because they had a lot in common regarding age groups, experiences, and physical, social, and psychological needs, and did not feel stigmatized. They felt they were allowed to relate more easily and freely with each other in adolescent HIV clinics. Yet the adult care providers, who don’t know or lack skills or an environment to provide care adolescents are accustomed to and feel they deserve, may lead to experiences of stigma r even discrimination. as exemplified by two respondents:

*“Yeah, we feel that and we think that’s what works for us because we feel we are the same age it’s easy to understand each other but in the adult clinics, adolescents fear the unfriendly environment where they may even meet there their relatives, their aunties their uncles, who may expose their HIV status outside”. (female, 15-19 years)”.*

### Fear of stigma from health care providers in adult clinics

The adolescents expressed fear for the health care providers in the adult clinic. The adolescents thought that working with the new providers would not be favourable to them and providers in the adult clinics may lack knowledge and skills of handling them, and unlike those in adolescent clinics, may make them feel stigmatized by the repetition of their life stories, as exemplified by three respondents:

*“It’s not okay because the stigma is high, discrimination, some of us are still in school so, we fear those, so we find that it’s hard for someone to be exposed outside in the adolescent clinic”. (female, 15–19 years)*

*“I fear to find different and new health providers in the adult clinic who do not know me and they don’t know my story”. (male, 15–19 years)*

*“Fear that the health workers in the adult clinic are not kind and caring as those in the adolescent clinic”. (female, 15–19 years)*

The adolescents expressed fear that if they went to the adult clinics, the adults would disclose their sero status and this would create stigma in the communities they live in

*“I fear to find relatives and village mates in the adult clinic who might disclose my HIV status back in the village to their children and other people in the village” (female, above 24 years).*

### Congestion and long waiting lines

Some of the adolescents who had visited the adult clinic expressed that adults spend a lot of time in the clinic from morning to evening, whereas in adolescents’ clinics they are seen very fast and they leave. The adult clinics have so many clients and are congested, Adolescents don’t want to spend a lot of time in the clinics

*“When I come putting on my uniform, they give me the medicine but there you have to wait until they finish those who came first but here, if I come putting on my uniform or even if I am not putting it on, I get my medicine fast”. (Male, 15–19 years)*

*“Some of us are schooling going children, some are working so, someone will escape from school to come to pick medications, some will escape from work to come to pick medications, so, when we are transitioned for real, remember when you join adulthood, then, for them they know once I am going for medication I am going to make all that day for medication but for us, we are always on a quick schedule. As you come you left school when having a test in the afternoon, you come rushing you say, aya ya, I am going for a test, they give you your medicine and you move but the adults stay here the whole day. We see, some of our parents we come with them and they expect to spend the whole day and you find you came with the parent for you you’re done but she is still there”. (female, 20–24 years)*

### Fear to lose friends

The adolescents expressed that if they are transferred to adult clinics they will lose their friends since they will be given different appointments whereas, in adolescent clinics they had a special day when they met as adolescents, this scares them a lot. Yet many adolescents had formed special bonds with other adolescents since the time they met in the pediatric clinics. They had shared experiences and dreams and formed special bonds, including getting treatment buddies from their age mates, which enriches their diverse experiences and coping skills. This view is exemplified by two adolescents:

*“…do not want to go to the adult clinic because they will miss their age mates since they usually come to the clinic and share their experiences”. (female, 15–19 years)*

*“I would not wish to go to the adult clinic, is because I will miss my friends. When you come here, you chat with this one and you have a different conversation with another person”. (male, 15–19 years)*

### Facilitators to transitioning

#### Preparation for transitioning is critical

The adolescents expressed that preparation is paramount for them to transition and it may hinder them from transitioning because they don’t know what to expect to do there and what is expected of them. This could be that they are not prepared well or they don’t know what to expect in adult clinics. Some adolescents think they are still young and that they have not reached that age of going to the adult clinic. Initially, the Ugandan guidelines said that the age of transitioning was 18 years and later moved it to 24 years. However, some clients are above 24 years still seen in the clinic

*“We don’t want to go to the adult clinic because they think they will be treated like adults yet they are still those vulnerable people who still need that care like that in the adolescent clinic”. (female, 15–19 years)*

*“I don’t want to go to the adult clinic because I didn’t know what I am going to do there”. (male, 15–19 years)*

### Moving as a cohort facilitates the transition

Adolescents expressed that taking them as a cohort to the adult clinic so that they move with their friends whom they have been with and are familiar with would facilitate the transitioning process instead of distributing them in the different adult clinic days or creating a different day for the transitioned adolescents in the adult clinic and not mixing them with the adults.

*“If they are to change us to the adult clinic, they should take us as a group because now can see your friends and age mates maybe they get like 10 adolescents and they take them there as a group but when you have been knowing each other. So, that helps”. (Male,20–24 years)*

*“…like all of us as we are here, all of us should go at once because as we are here, we know our selves and we associate. So, even if they give us one day in a month, but we are as we are here when we are age mates but not sitting here next to a 70 year, grand mum”. (Female 15–19 years)*

### Transitioning preparation is key to a successful transition

Preparing the adolescents earlier before being transitioned to the adult clinic, like first talking to them about transitioning and telling them everything about the adult clinic would facilitate transitioning. Such sessions may include information on the importance of transitioning, how adolescents and parent may prepare to transition, what they are likely to experience after transitioning, and how the transition will be handled, as exemplified by two respondents:

*“We should be having sessions with parents and the health workers and discuss with them to on how to treat the adolescents well when they are transitioned to the adult clinic, …not to be judgemental, not to disclose their status in the village, not to talk about them, not to discriminate …the adolescents among others such that the adolescents feel comfortable when they go to the adult clinic”*. (female, 15–19 years)

*“I think transitioning should be introduced to us from the point we step in and become their client so that we grow up with that in mind, (so that) it’s not like an ambush, like the way they are doing it now. But if at a point we steeped in here during counselling, they added that point of transitioning each time I have a counselling session they tell it to me, it wouldn’t be new to me and I will be feeling comfortable going there because they will be telling me the advantages and why but now it’s had for someone”. (Female, 20–24 years)*

### Care in the adult clinic offers new opportunities

The adolescents want to be treated the way they have been treated in the adolescent clinics as they move to adult clinics and felt that could facilitate their transitioning. All the ART clinics had peer support groups and some of the facilities were implementing the new program (YAPS) from the Ministry of Health. In peer support groups adolescents to help each other to improve and better manage their situation, share challenges, and discuss solutions. Members support each other to implement decisions made to meet their psychological, social, physical, and medical needs. Unfortunately, most adult HIV clinics lack formal peer support services, which makes transitioning a daunting task and a process filled with a lot of fear of the unknown and loss of peer support. However, others perceived this as an opportunity to forge and nurture new friendships:

*“I feel like I still need more help (as in the adolescent clinic) from my peers (peer support) through their support groups and also from health care providers especially counsellors and social support on adherence to medication among other challenges they face”. (Female, 20-24 years).*

Others felt they should be transitioned to continue with health workers who knew about them following them in the adult HIV clinics. Besides, privileges like being fast-tracked when health workers recognize them as students, providing incentives like tea and snacks, and shortening their facility waiting times, would facilitate a successful transition, as exemplified by three adolescents:

*“They should provide patients in adult clinics with the same privileges as those in the adolescent clinic, for example, giving them porridge, having adequate counsellors, short waiting time among others”. (female 15–19 years)*

*“Treating adolescents well like children even when they are transitioned to the adult clinic, like being caring and kind to them while in the adult clinic”. (Male,15–19 years)*

*“Moving with the same health providers to the adult clinic whom the adolescents are used to and who knows more about them”. (Female, 15–19 years)*

Some adolescents were ready and eager to move to the adult HIV clinics because of some of the benefits they will get the moment they get into adult clinics as compared to adolescent clinics, which at the moment do not have such services. Such opportunities included meeting new people, making new friends, and getting new ideas from adults living with HIV.

*“For me, I would love to go to the adult clinic, such that I can meet adults with beneficial ideas and knowledge, and also to have sensible and mature conversations with them” (female, 20–24 years)*

Such benefits included counselling and services for sexual and reproductive health, including family planning and information on safe sex, information on employment and life skills, and access to different forms of ART services delivery that are not available in the paediatric and adolescent clinics, and utilization of information delivery models such as social media platforms. Other benefits include service delivery models where patients may be supported with home delivery of drugs. Ensuring the availability of such services could facilitate the transition to adult HIV clinics:

*“I would love to go to the adult clinic because now when I get there obviously there are packages that are given in the adult clinic that I can’t get here like practicing safer sex, family planning and by that time I will be engaged so they will be beneficial to me” (Female,20-24 years).*

*“Differentiated Service Delivery model, they have privileges of getting drugs from home, in the community they don’t have to come here and for the adolescents, it’s the clinic and I would also love to be on those groups where you don’t have to come to the clinic, I only have to come to the clinic when I have issues” (Male,20–24 years)*

Participants who were eager to transition demonstrated a more positive perspective and outlook at benefits and new opportunities after transitioning, and therefore more understanding and acceptance of their disease, which indicates high self-esteem, confidence, and lack of stigma.

## Discussion

There is a need to investigate the barriers and facilitators of the successful transition from adolescent to adult HIV care, as these impact on adherence or continuation of lifelong ART, drug side-effects, retention in care, disclosure of serostatus, and mental health challenges associated with living with a stigmatizing chronic disease**.** The barriers for transitioning included; Unfriendly adults in adult clinics, Care provided in the adolescent clinics, fear of stigma from health care providers, Congestion and long waiting time, fear to lose friends were barriers to transitioning. Transitioning preparation is key to a successful transition**,** moving as a cohort facilitates transition, and care in adult clinics offers new opportunities were perceived as facilitators of the readiness and successful transitioning.

Adolescent-friendly services are accessible, acceptable, appropriate, effective, and equitable [20]. Providers of these services are sensitive to their young clients’ needs, they encourage autonomy and demonstrate respectful and non-judgmental attitudes. In this study, one of the barriers to transitioning was the creation of adolescent clinics. The care provided in the adolescent clinics was very satisfactory to the adolescents and they didn’t see the need to transfer to adult clinics. Adolescent HIV care is characterized by an emphasis on multidisciplinary on-site care with a youth friendly environment, a family-centered focus, and psychosocial support which attends to adolescent developmental needs [21]. Many adolescents with HIV (both perinatal and behaviourally acquired) develop strong and longstanding relationships with their care teams, often seeing them as members of their extended family, especially in the context of parental loss [22, 23] As such adolescents may be reluctant to disengage from health care providers in the adolescent clinics and likewise. Integrating adolescent friendly days or clinics in ART care has shown to improve retention in care [24] which improves health outcomes. However, most of these adolescents and young people have grown and need to move to adult clinics to create space for those patients in pediatrics clinic. Does this pose a question could the creation of adolescent clinics hinder their transitioning? The transition should be a gradual process of preparing and supporting the adolescent to make the shift from dependence on caregivers to self-management and autonomy, and into more developmentally and medically appropriate care. A transition support plan should be started as soon as the adolescent is disclosed to and it should be individualized transition plan which should be flexible to meet the adolescent’s needs.

The engagement of adults in adult ART clinics to support adolescents in transitioning cannot be underestimated. In this study, most adolescents saw unfriendly adults in adult clinics as a barrier to transitioning. It is important to explain the transitioning process and its importance to the adults in adult clinics so that they can support these adolescents. The role of caregivers and adults has been documented in many studies family caregivers wanted early knowledge about transition; these individuals are an important resource to find potential solutions to guide the transition process [25, 26]. It is important to ensure that the adolescents can be accommodated in the clinic they are being transferred too. This includes the setup of the clinics including the adult’s psychosocial support can help adolescents as well as their caregivers gain confidence in themselves and their coping skills. It can increase patients’ understanding and acceptance of comprehensive HIV care and support services, encourage adherence to HIV treatment, and equip them with skills to make informed secondary prevention decisions. Such support can also help prevent adolescents living with HIV from adopting risk-associated behaviours or from developing more severe mental health problems. Caregivers and adults also benefit from the support that acknowledges the stress they are under and validates their concerns about their children while enabling them to learn how to cope with the adolescent’s developmental and health needs.

HIV is a highly stigmatized illness and many adolescents and young people living with HIV face HIV-associated stigma and disclosure to sexual partners, friends, and family is a barrier to engagement in adult care [27, 28] In this study, adolescents expressed fear that if they went to the adult clinics, the adults would disclose their sero status and this would create stigma in the communities they live in. Adults, parents, and caregivers need to understand that stigma can affect an adolescent’s ability to live positively with HIV and affects an individual’s sense of self-worth and self-esteem, the ability to seek emotional and psychosocial support through disclosure to others, the confidence to adhere to treatment at school or in the workplace, and the willingness to seek health services on continually [29]. Understanding and learning how to deal with stigma is one of the skills that adolescents living with HIV need to acquire as they move into adulthood. There is a need to address stigma by creating clinic wide strategies to eliminate stigma towards adolescent patients in the clinical setting [29].

One of the barriers mentioned was congestion and long waiting hours in adult clinics. The HIV service delivery approach for adolescents usually Fast-track drug pickup and has flexible clinic hours that take care of both in-school and out of school adolescents so they don’t wait a lot. Adult HIV clinics are often more formal with limited scheduling flexibility, more patient-and disease-focused care, less co-located specialty care, and fewer youth-friendly services [11] These characteristics may explain the poor outcomes of ALHIV seen in adult care [30]. Adolescents and young people transitioning to adult clinics have identified fear of the adult clinic environment as a barrier to smooth and proper transition and have described difficulties after transfer to adult clinics in dealing with congestion and longer wait times [23, 25]. Engaging and training adult providers in adolescent-friendly HIV care models may be useful as many adult providers lack the expertise or will to provide youth-friendly services in the adult setting [22, 31].

Most of the adolescents have grown in these ART clinics and they are very comfortable in these clinics with their peers and their providers. The study showed that the adolescents wanted to be moved as a cohort or at least 10 of them on a specific day in the adult clinic. Separation from the group they have known is a challenge a study done in the US found out that most of the adolescents felt like they had lost a family when they talked about transitioning [26]. It is important to assist adolescents to identify barriers (real or perceived) to the transitioning process so that the providers, caregivers, and adolescents can explore potential strategies to overcome them.

The transition needs to be carefully planned and managed, taking into consideration the adolescent’s medical, psychological, and social needs. Studies have indicated that a clearer transition process, with collaborative planning among youth and health-care teams, time to prepare for the transition, individualized services, and supportive process that involves both paediatric/adolescent and adult healthcare providers and respects the individual patient’s readiness to transfer care [23, 26, 32]. The transition should be a gradual process of preparing and supporting the adolescent to make the shift from dependence on caregivers to self-management and autonomy, and into more developmentally and medically appropriate care. Carefully planned transition recognizes the evolving developmental, medical, emotional, educational, and social needs. In this study, it showed that transition preparation acted as a barrier for effective transitioning if it was not done well and a facilitator if it was done well.

Peer support is about giving and receiving help to others with respect, shared responsibility, and mutual agreement. It is about understanding another’s situation empathically through the shared experience of emotional and psychological pain. Studies have shown that peer support groups lead to improvement of adolescent emotional and physical wellbeing and it positively influences medical outcomes [33, 34] Peer support groups are groups of people who come together because they share a common situation. In this study adolescents found the Young adolescent program Support (YAPS) Could facilitate readiness and transitioning. In peer support groups adolescents found out that they help each other to improve and better manage their situation, share challenges, and discuss solutions. Members support each other to implement decisions made to meet their psychological, social, physical, and medical needs. There is a need to use peer and young groups in transitioning and maybe they could be used to receive these young people in the adult clinics so that when they transition they see familiar faces that could improve the transitioning process.

### Study limitations

The participants were selected by the peer leader from the ART clinic this could have posed a selection bias. But while all adolescents and youth were eligible, we wanted, through maximum variation sampling, to have a representation of all age groups among the adolescents and youth. We also wanted a representation of the diverse experiences of the different adolescents and youth to be represented. While this may have introduced selection bias by choosing to approach consciously or unconsciously those whom they felt would be a good candidate for the focus group, all patients had more or less equal opportunity to be presented, as there were no other selection criteria which could have led to exclusion of some patients. First, all participants in the study were recruited from adolescent clinics, so we do not have a representation of views and perceptions of adolescents and youth in the adult clinics. Their experiences (as facilitators or barriers) may be less generalizable to all adolescents and YLWH who lack access to paediatric, adolescent, or antenatal care. The experiences of in-care and out-of-care patients may differ markedly. Besides, the study included participants who were infected perinatally as well as those whose mode of infection was unknown or was behaviorally infected. The populations may be different, and those infected perinatally may have differing opinions to those infected through high-risk behaviour, though for this study sample themes were consistent across transition groups. Also, no views of adolescents in adult HIV care were obtained. Even then, there were no interviews conducted with health care providers, patients, or caregivers to corroborate the information provided.

## Conclusion

Adolescents and young people did not want to transition to adult clinics mainly because of the supported care provided in the adolescent clinic. A range of individual, social, and health systems and services hinders transitioning readiness and transitioning. Understanding barriers and facilitators can enable the Ministry of Health to improve the quality of life of adolescents and young people through linkage to care, adherence, retention, and viral suppression. There is a need to better planning and preparation for clinical providers and young people with a focus on age-appropriate and individualized case management transition. Furthermore, to address stigma by creating clinic wide strategies to eliminate stigma towards adolescent patients in the clinical setting by engaging them planning, service delivery and evaluation of the services.

## Data Availability

The acquired and/or analyzed data are not publicly available because of the lack of authorisation from the children’s legal guardians, and the agreement with the Research Ethics Committee that the database would remain with the corresponding author only. However, all data can be made available by the corresponding author upon reasonable request.

## Abbreviations

ALHIV: Adolescents living with HIV
ART: Antiretroviral Therapy
PHIV: Perinatally HIV
HIV: Human Immunodeficiency Virus
HC: Health Center
HTC: Health Transiting Clinics
MOH: Ministry of Health
PNFP: Private Not for Profit
RRH: Regional Referral Hospital
YAPS: Young adolescent Program Support
YPLHIV: Young people living with HIV
